# Risk of bias in routine mental health outcome data: the case of Health of the Nation Outcome Scales

**DOI:** 10.1136/bmjment-2025-301669

**Published:** 2025-06-05

**Authors:** Edward Penington, Ryan Williams, Apostolos Tsiachristas

**Affiliations:** 1 Department of Psychiatry, University of Oxford, Oxford, UK; 2 Department of Brain Sciences, Imperial College London, London, UK; 3 South London and Maudsley NHS Foundation Trust, London, UK; 4 Department of Primary Care Health Sciences, University of Oxford, Oxford, UK

**Keywords:** Data Interpretation, Statistical, Schizophrenia & psychotic disorders, Depression & mood disorders, Adult psychiatry

## Abstract

**Background:**

Routine outcome data in secondary mental health services have significant potential for service planning, evaluation and research. Expanding the collection and use of these data is an ongoing priority in the National Health Service (NHS), but inconsistent use threatens their validity and utility. If recording is more likely among certain patient groups or at specific stages of treatment, measured outcomes may be biased and unreliable.

**Objective:**

The objective is to assess the scale, determinants and implications of incomplete routine outcome measurement in a secondary mental health provider, using the example of the widely collected Health of the Nation Outcome Scores (HoNOS).

**Methods:**

A retrospective cohort study was conducted using routine HoNOS assessments and episodes of care for patients receiving secondary mental healthcare from an NHS Trust in Southeast England between 2016 and 2022 (n=30 341). Associations among demographic, clinical and service factors, and rates and timings of HoNOS assessments were explored with logistic regressions. Relationships between total HoNOS scores and related mental health outcomes (costs, relapse and improvement between assessments) were estimated after adjusting for the likelihood of assessment.

**Findings:**

66% of patients (n=22 288) had a recorded HoNOS assessment. Of the distinct episodes of care for these patients (n=65 439), 43% (n=28 170) were linked to any assessment, 25% (n=16 131) were linked to an initial baseline assessment, while 4.7% (n=3 094) were linked to multiple HoNOS assessments, allowing for evaluation of clinical progress. Likelihood and timing of assessment were significantly associated with a range of factors, including service type, diagnosis, ethnicity, age and gender. After adjusting for observed factors determining the likelihood of assessment, the strength of association between HoNOS scores and overall costs was significantly reduced.

**Conclusion:**

Most of the activity observed in this study cannot be evaluated with HoNOS. HoNOS assessments are highly unlikely to be missing at random. Without approaches to correct for substantial gaps in routine outcome data, evaluations based on these may be systematically biased, limiting their usefulness for service-level decision-making.

**Clinical implications:**

Routine outcome collection must increase significantly to successfully implement proposed strategies for outcome assessment in community mental healthcare without inconsistent records undermining the use of resulting data.

**WHAT IS ALREADY KNOWN ON THIS TOPIC**

- Routinely collected outcome data can be effective in guiding individual care and shared decision-making, but it is also extremely useful for improving and demonstrating the effectiveness and value of mental health services. Complete paired assessments, including baseline and progress measurements at every care contact, are crucial to making the most out of routine outcome data. The National Health Service England is expected to implement new strategies for outcome assessment, including wider use of patient-reported outcome measures, across community mental health services.

**WHAT THIS STUDY ADDS**

- This study shows the scale of the challenge facing the adoption and implementation of new routine outcome measurement in practice. The overwhelming majority of secondary mental healthcare currently provided cannot be directly evaluated using routine data. Where partial routine outcome data is used to assess service quality or resource needs, it is likely to be biased by both observed factors (which can be adjusted for) and unobserved factors (which cannot).

**HOW THIS STUDY MIGHT AFFECT RESEARCH, PRACTICE OR POLICY**

- The adoption of novel approaches to outcome assessment in community mental healthcare should focus on improving consistency and coverage of outcome measure recording to mitigate the risks to the value of data. However, policy-makers should also be aware that 100% coverage is unlikely to be achieved in practice and should prepare explicit methods to understand and correct for bias in incomplete routine outcome data.

## Background

 The National Health Service (NHS) in England places a heavy and growing emphasis on routine outcome data in secondary mental healthcare. It has recently recommended the routine use of three new outcome measures—DIALOG, the Goal-Based Outcomes tool and Recovering Quality of Life 10-item scale—in community mental health services for adults with severe mental illness.^1^

These new measures are intended to replace the Health of the Nation Outcome Scores (HoNOS),^2^ an outcome measure designed to capture symptom severity and associated impairments in functioning. While it is now being phased out,^1^ it has been the most widely used and collected routine outcome measure in NHS mental health services in England due (in part) to its role in reimbursement as part of the Mental Health Clustering Tool.^3 4^ These standardised measures are intended to inform patients and practitioners in shared decision-making at the individual level, but also to be used as common outcome measures at the service level to demonstrate effectiveness, improve service quality, identify care needs and inform value for commissioners.

While research has been undertaken on the clinical impacts of routine monitoring and feedback in primary and secondary mental health care,^5 6^ there has been less research on how best to use this data at the service level. As shown in the use of outcome measurement in NHS Talking Therapies for anxiety and depression, ‘complete paired data’ measuring outcomes before, during and after therapy is necessary to accurately capture the impacts of treatment in routine data.^7^ This poses a particular challenge in severe mental illness, where it may be challenging to establish a clear baseline when conditions and therapies are overlapping. If the drivers of missing outcomes data are related to the outcome of interest—for example, if patients are more likely to be assessed (or less likely to engage with assessment) at certain points in their treatment when outcomes may be better or worse—then service-level analysis of that data will be biased and risk undermining evaluations of service effectiveness and allocation of resources. While improving the completeness and consistency of outcome recording will help address these issues, it is also important to understand the drivers and implications of incomplete outcome data in practice to ensure their use in service evaluation, planning and commissioning is not undermined by statistical bias.

## Objective

The aim of this study is to use data previously collected by secondary mental health services to understand patterns in missing outcome data and its potential impact on statistical relationships used in mental health services research and administration.

## Methods

An analysis was undertaken using referrals to community and inpatient mental healthcare to explore the observable factors associated with patients being assessed with HoNOS at different points during the referral pathway. To test whether this pattern of missing data influenced the interpretation of outcome data, the statistical relationship between total HoNOS Score and other outcomes of interest was then tested with and without correcting for observable drivers of missing data.

### Setting and data source

Electronic patient records were obtained for adults on the caseload of Oxford Health NHS Foundation Trust (OHFT) between March 2016 and August 2022. OHFT provides publicly funded secondary mental healthcare for a large catchment area of around 1 million people in South East England (mainly the counties of Oxfordshire and Buckinghamshire). Data were obtained in the form of episodes of care (ie, periods from referral to discharge) with services provided by OHFT, including crisis response teams, psychiatric inpatient and emergency department services and psychiatric liaison services (specialist mental health teams providing in-reach services for patients in general hospitals). Episodes with a community mental health team (CMHT) or specialist early intervention in psychosis (EIP) service with at least one attended in-person or telephone contact were also included. Episodes with services providing treatment across community and inpatient settings (eg, psychological therapy teams), or disorder-specific services not using HoNOS (eg, eating disorder teams) were excluded.

### Definition of variables

#### Service user characteristics

Month and year of birth, gender and residence at time of data extraction were recorded for most of the study population. Age was calculated at the start of each episode. Socioeconomic deprivation was proxied using the Index of Multiple Deprivation 2019 for lower-level super output area of residence, geographic units of around 1500 people which are the smallest statistical areas for which deprivation indices are calculated in England.^8^ Mental health diagnosis was extracted based on recorded International Statistical Classification of Diseases and Related Health Problems code Tenth Revision (ICD-10)—the standard diagnostic manual for psychiatric practice in the UK—where available. As a substantial number of records did not have a recorded diagnosis code, the diagnosis of a psychotic disorder was therefore inferred based on both recorded diagnosis and prior contact with or referral to psychosis-specific services. Information on ethnicity was recorded for most patients and included in analysis as explicit recording of black, Asian or minority ethnic (BAME).

#### HoNOS assessments

Likelihood of assessment was estimated at the episode level. An episode was considered to have any matched HoNOS assessment where at least one valid assessment was recorded between the recorded episode start date (acceptance onto caseload) and 6 months following the first recorded contact. 6 months is the maximum recommended time between HoNOS assessments under NHS Payment by Results.^3^ An episode was considered to have multiple HoNOS assessments if more than one assessment during that episode met the same criteria, and an initial HoNOS assessment if an explicitly-coded initial assessment occurred at any date from acceptance onto the caseload up to and including the first recorded treatment. Matching by date and patient identifier alone was necessary as HoNOS assessments were not systematically recorded with episode or service identifiers.

When conducting analysis at the episode level, an assessment could be matched to more than one overlapping episode at multiple locations for the purposes of assessing the likelihood of assessment. When conducting analysis at the HoNOS assessment level, the associated episode was considered to be the one that began most recently before the assessment date. Associations between HoNOS and other outcomes used the adjusted total HoNOS Score. Each HoNOS assessment consists of scores between 0 (no problem) and 4 (severe to very severe problem) for 12 scales of health and social functioning. The adjusted total score was calculated out of a total possible score of 48, with missing items imputed from the mean of non-missing items.

#### Other mental health outcomes

The following outcomes were compared with HoNOS scores in the 6 months following assessment: episode-specific costs, overall costs, relapse, and overall improvement HoNOS Score. Costs were calculated from all recorded contacts, inpatient admissions and bed days for each patient. Unit costs for each contact, initial admission and bed day were taken from the Personal Social Services Research Unit (PSSRU).^9^ Episode-specific costs were calculated from contacts and admissions with the same location as the episode during the referral period up to 6 months following assessment, while overall costs included all services provided by OHFT in the same period. Relapse was defined as admission to an inpatient setting or contact with a crisis resolution and home treatment team (specialist outreach services which provide intensive treatment to patients in the community who would otherwise require inpatient care). This outcome was chosen due to its being observable in OHFT data with substantial and well-established implications for quality of life^10^ and resource use^11^ that applies across conditions assessed by HoNOS and has previously been used in a study of EIP services provided by OHFT.^12^ Overall improvement in HoNOS Score is defined as any reduction in adjusted total HoNOS Score between the first assessment and the last within the 6-month window. Respectively, these measures proxy the use of HoNOS for allocating resources within providers, reimbursement for providers based on HoNOS-clusters and the use of improvement as a measure of service-level outcomes and quality.

### Statistical analysis

#### Descriptive statistics

The demographics and overall service use of the study population are described by the presence of any recorded HoNOS assessment during the study period. Descriptive statistics at the episode level on the number of matched HoNOS assessments, duration of episodes, and episode-specific service user characteristics are reported by location type. Differences in categorical variables and differences in continuous variables were tested for statistical significance with Pearson’s χ^2^ tests and Wilcoxon rank-sum tests respectively.

#### Factors associated with HoNOS assessment

For each episode, the likelihoods of any matched HoNOS assessment, multiple HoNOS assessments and an initial HoNOS assessment were estimated separately using logistic regression, controlling for the service type, episode duration and patient characteristics. SEs were clustered at the patient level. Each regression was used to calculate the predicted probability of an episode resulting in an HoNOS assessment.

#### Relationship of HoNOS with mental health outcomes

At the HoNOS assessment level, the following relationships between HoNOS Score and outcome were estimated:

1. Overall costs (ie, from any OHFT service) in the 6 months following any HoNOS assessment.
2. Costs in the first 6 months of an episode following an initial HoNOS assessment (ie, only those associated with a specific referral to a service).
3. Relapse in the 6 months following any HoNOS assessment.
4. Improvement in scores between multiple HoNOS assessments.

Each of these regression models was estimated with generalised linear models, controlling for patient demographics, service type and clinical characteristics. Cost was estimated as a Gamma regression with a log link. Relapse likelihood and rate of improvement were estimated with logistic regression (binomial with logit link). In all models, SEs were clustered by patient.

To assess the extent of bias arising from inconsistent HoNOS assessment in these relationships, we specified the following regression models: with only an intercept and HoNOS Score term (unadjusted); inverse probability-weighted for the likelihood of assessment, using calculated predicted probabilities (inverse probability weighting (IPW)); controlling for observed individual-level and episode-level factors as covariates (adjusted); and both doubly robust correction with both IPW and factors as covariates (IPW adjusted). Results were reported as the exponentiated coefficient of HoNOS Score (adjusted OR or mean ratio (MR)).

## Findings

### Descriptive statistics

Table 1 below summarises the characteristics of individuals with at least one service use episode meeting the inclusion criteria during the study period.

**Table 1:** Patient-level descriptive statistics

	Overall (n=30 341)	Any HoNOS recorded (n=22 288)	No HoNOS recorded (n=8053)	P value
Mean episodes included in study (SD)	2.16 (2.39)	2.43 (2.65)	1.41 (1.10)	<0.001*
Any matched HoNOS assessment, % (n)	66% (20 152)	90% (20 152)	NA	<0.001†
Duration of follow-up in months, mean (SD)	13.47 (18.55)	16.75 (19.76)	4.42 (10.20)	<0.001*
Age in years at first episode, mean (SD)	36.69 (15.00)	37.45 (15.26)	34.60 (14.02)	<0.001*
Female, % (n)	54% (16 502)	55% (12 354)	52% (4148)	<0.001†
Ethnicity				<0.001†
Black and minority ethnic, % (n)	4.8% (1449)	5.6% (1243)	2.6% (206)	
Not recorded, % (n)	44% (13 334)	36% (7987)	66% (5347)	
Other, % (n)	0.9% (282)	1.0% (212)	0.9% (70)	
White, % (n)	50% (15 276)	58% (12 846)	30% (2430)	
Resident in most deprived quintile, % (n)	5.9% (1787)	5.6% (1250)	6.7% (537)	<0.001†
Recorded psychosis diagnosis (ICD10 F20.X–F29.X), % (n)	8.4% (2560)	11% (2344)	2.7% (216)	<0.001†
Recorded contacts, mean (SD)	21.42 (43.76)	27.19 (48.81)	5.45 (16.52)	<0.001*
Recorded inpatient bed days, mean (SD)	14.83 (83.01)	19.65 (96.11)	1.47 (12.57)	<0.001*
Inpatient or crisis team episodes (relapse), mean (SD)	0.50 (1.20)	0.58 (1.34)	0.28 (0.59)	<0.001*
Cost, mean (SD)	£10 299 (34 156)	£13 358 (39 225)	£1833 (6292)	<0.001*

Note that p values represent test of difference between HoNOS recorded and no HoNOS recorded.

* Wilcoxon rank-sum test.

† Pearson’s χ2 test.

HoNOS, Health of the Nation Outcome Scores; ICD10, International Statistical Classification of Diseases and Related Health Problems code Tenth Revision; NA, not applicable.

The study sample consists of 30 341 individuals, of whom 54% (n=16 502) were women and 4.8% (n=1449) were recorded as black or minority ethnic. 73.5% of individuals (n=22 288) had at least one HoNOS assessment recorded during the study period. Of those, 90% had at least one HoNOS assessment matched to an included episode. Individuals with any HoNOS recorded had a mean age of 37.45 (SD: 15.26), compared with 34.60 (SD: 14.02) for those without. A higher proportion of those with HoNOS recorded were women (55%, n=12 354 vs 52%, n=4148) and had a recorded diagnosis of psychosis (11%, n=2344 vs 2.7%, n=216). 5.6% (n=1250) of those with HoNOS and an address recorded lived in the most deprived areas, compared with 6.7% (n=537) for those without. Individuals with HoNOS had considerably more recorded service use of every kind, leading to an average cost over the study period of £13 358 (SD: 39 225), compared with £1833 (SD: 6292) for those without an HoNOS Score.

Table 2 summarises the variation in HoNOS assessments by episode (n=65 439) by the service type.

**Table 2:** Episode-level descriptive statistics

	Overall (n=65 439)	CMHT (n=32 964)	CRHT (n=7404)	EIP (n=1767)	Inpatient (n=7876)	PLS (n=15 428)	P value
Initial HoNOS assessment, % (n)	25% (16 131)	42% (13 796)	20% (1450)	21% (373)	5.2% (409)	0.7% (103)	<0.001*
Any HoNOS assessment, % (n)	43% (28 170)	65% (21 585)	38% (2827)	55% (968)	32% (2509)	1.8% (281)	<0.001*
Multiple HoNOS assessments, % (n)	4.7% (3094)	5.6% (1854)	5.8% (433)	12% (208)	7.5% (588)	<0.1% (11)	<0.001*
Episode duration in weeks, mean (SD)	7.15 (9.25)	10.63 (9.97)	3.87 (5.49)	17.40 (11.01)	6.22 (7.31)	0.60 (2.95)	<0.001†
Age in years at episode start, mean (SD)	38.34 (15.68)	38.41 (15.52)	36.56 (13.32)	31.16 (11.60)	44.49 (17.18)	36.74 (15.72)	<0.001†
Prior diagnosis of psychosis, % (n)	10% (6676)	6.8% (2249)	7.2% (530)	13% (238)	33% (2620)	6.7% (1039)	<0.001*

Note that p values represent test of difference between different service types.

* Pearson’s χ2 test.

† Kruskal-Wallis rank-sum test.

CMHT, community mental health team; CRHT, crisis resolution and home treatment team; EIP, early intervention in psychosis; HoNOS, Health of the Nation Outcome Scores; PLS, psychiatric liaison service.

Overall, 25% (n=16 131) of episodes included an initial HoNOS assessment (ie, on or before the first recorded contact). 43% (n=28 170) of all episodes received any HoNOS assessment during the first 6 months following contact, and 4.7% (n=3094) were linked to multiple HoNOS assessments within the same period, allowing for evaluation of clinical progress. There was variation in the overall level of assessment—CMHTs were more likely to record assessments than average by any measure—as well as variation in the pattern of assessment—inpatient episodes were less likely to result in any HoNOS assessment but more likely to result in multiple HoNOS assessments.

### Factors associated with HoNOS assessment

Table 3 shows the results of separate logistic regression models estimating the likelihood of an episode involving an assessment with HoNOS.

**Table 3:** Factors associated with HoNOS assessment at episode level

	Any HoNOS assessment		Initial HoNOS assessment		Multiple HoNOS assessments	
	Adj. OR	(95% CI)	Adj. OR	(95% CI)	Adj. OR	(95% CI)
(Intercept)	1.87	(1.68 to 2.08)	0.72	(0.64 to 0.80)	0.04	(0.03 to 0.05)
Service type: (ref: CMHT)						
CRHT	0.33	(0.32 to 0.35)	0.34	(0.32 to 0.36)	1.19	(1.07 to 1.33)
EIP	0.68	(0.61 to 0.75)	0.38	(0.33 to 0.43)	2.82	(2.40 to 3.30)
Inpatient	0.26	(0.24 to 0.27)	0.09	(0.08 to 0.10)	1.01	(0.89 to 1.14)
PLS	0.01	(0.01 to 0.01)	0.01	(0.01 to 0.01)	0.01	(0.01 to 0.02)
Resident in most deprived quintile	1.06	(0.97 to 1.16)	1.06	(0.97 to 1.16)	0.92	(0.76 to 1.11)
Prior diagnosis of psychosis	0.68	(0.63 to 0.73)	0.38	(0.34 to 0.42)	1.07	(0.93 to 1.23)
Ethnicity category: (ref: BAME)						
Not known	0.93	(0.85 to 1.02)	1.18	(1.07 to 1.30)	0.55	(0.47 to 0.66)
Other	1.00	(0.79 to 1.28)	0.96	(0.75 to 1.23)	1.02	(0.70 to 1.49)
White	0.97	(0.89 to 1.06)	1.19	(1.08 to 1.31)	0.67	(0.57 to 0.78)
Age category: (ref: 18–24)						
25–34	0.97	(0.92 to 1.03)	0.99	(0.94 to 1.05)	1.17	(1.02 to 1.33)
35–44	0.97	(0.91 to 1.03)	0.93	(0.88 to 1.00)	1.19	(1.03 to 1.37)
45–54	0.96	(0.90 to 1.02)	0.94	(0.88 to 1.00)	1.28	(1.11 to 1.47)
55–64	1.04	(0.96 to 1.12)	0.86	(0.79 to 0.93)	1.71	(1.47 to 1.99)
65+	1.46	(1.32 to 1.61)	0.59	(0.53 to 0.66)	7.39	(6.37 to 8.58)
Female	1.25	(1.20 to 1.30)	1.10	(1.05 to 1.15)	1.45	(1.33 to 1.58)
Year: (ref: 2016)						
2017	1.11	(1.04 to 1.19)	1.14	(1.06 to 1.22)	1.16	(1.00 to 1.34)
2018	0.98	(0.92 to 1.05)	1.08	(1.01 to 1.17)	1.16	(1.00 to 1.34)
2019	1.11	(1.04 to 1.19)	1.19	(1.11 to 1.28)	1.06	(0.91 to 1.24)
2020	0.81	(0.75 to 0.87)	0.7	(0.66 to 0.77)	1.22	(1.05 to 1.42)
2021	0.81	(0.76 to 0.87)	0.58	(0.53 to 0.62)	1.53	(1.33 to 1.76)
2022	0.94	(0.74 to 1.19)	0.68	(0.51 to 0.91)	1.30	(0.80 to 2.10)
N	65 439		65 439		65 439	
AIC	66 855.56		57 009.03		22 061.86	
BIC	67 055.51		57 208.99		22 261.82	
Pseudo R^2^	0.39		0.32		0.14	

Note that SEs are heteroskedasticity robust and clustered by individual.

Adj. OR, adjusted odds ratio; AIC, Akaike information criterion; BAME, black, Asian or minority ethnic; BIC, Bayesian information criterion; CMHT, community mental health team; CRHT, crisis resolution and home treatment team; EIP, early intervention in psychosis; HoNOS, Health of the Nation Outcome Scores; N, Number of observations; PLS, psychiatric liaison service.

Episodes with all other service types were less likely to result in any HoNOS assessment than CMHTs (the reference category), most notably psychiatric liaison services with an adjusted OR of 0.01 (95% CI: 0.01 to 0.01). Of the other services included, EIP teams were most likely to be associated with any assessment at an adjusted OR (adj. OR) of 0.68 (95% CI%: 0.61 to 0.75). A similar pattern is observed in the models of initial HoNOS assessment; however, episodes with crisis teams (adj. OR 1.19; 95% CI: 1.07 to 1.33) and EIP teams (adj. OR 2.82; 95% CI: 2.40 to 3.30) were more likely to result in multiple HoNOS assessments within an episode. Episodes where a patient had a diagnosis of psychosis were less likely to result in any assessment (adj. OR 0.68; 95% CI: 0.63 to 0.73), or in an initial assessment (adj. OR 0.38; 95% CI: 0.34 to 0.42). There is no evidence of difference in recording of HoNOS by deprivation (adj. OR 1.06; 95% CI: 0.97 to 1.16). White patients were more likely to receive an initial assessment (adj. OR 1.19; 95% CI: 1.08 to 1.31), but less likely to receive multiple assessments (adj. OR 0.67; 95% CI: 0.57 to 0.78) compared with those recorded as BAME. Women were more likely to be assessed with HoNOS at any point (adj. OR 1.25; 95% CI: 1.20 to 1.30), as well as more likely to receive initial assessments (adj. OR 1.10; 95% CI: 1.05 to 1.15) and multiple assessments (adj. OR 1.45; 95% CI: 1.33 to 1.58). Both age at the start of the episode and time-varying fixed effects had varying effects on the rates of initial, overall and repeat HoNOS assessments, particularly for older patients (over 65) and during the COVID-19 pandemic (2020 and 2021), respectively.

### Association of HoNOS with other outcomes and service use

Table 4 summarises the models predicting overall cost, episode-specific cost, relapse incidence and improvement in overall score in the 6 months following an HoNOS assessment, showing the OR or MR associated with a single unit increase in total HoNOS Score. The full results of each model are provided in tables A1–A4 in the online supplemental material, respectively.

**Table 4:** Modelled outcomes and association with total score following HoNOS assessment

		Unadjusted		Adjusted	
		Unweighted	IPW	Unweighted	IPW
Overall cost following any assessment	MR	1.08	1.08	1.06	1.05
	(95% CI)	(1.08 to 1.09)	(1.07 to 1.09)	(1.06 to 1.06)	(1.05 to 1.06)
Episode cost following initial assessment	MR	1.09	1.09	1.06	1.04
		(1.08 to 1.10)	(1.06 to 1.11)	(1.05 to 1.06)	(1.03 to 1.05)
Relapse following any assessment	OR	1.07	1.07	1.06	1.06
		(1.07 to 1.08)	(1.06 to 1.08)	(1.06 to 1.07)	(1.04 to 1.07)
Improvement between multiple assessments	OR	1.15	1.12	1.16	1.15
		(1.13 to 1.16)	(1.07 to 1.18)	(1.14 to 1.17)	(1.10 to 1.21)

Note that results are shown with and without adjusting for observed episode characteristics (service type, deprivation, prior diagnosis of psychosis, ethnicity, age, gender and year), and with and without inverse probability weighting (IPW) for the likelihood of relevant assessment, or assessments, occurring based on the episode it occurred within. SEs are heteroskedasticity robust and clustered by individual.

HoNOS, Health of the Nation Outcome Scores; MR, (adjusted) mean ratio; OR, (adjusted) odds ratio.

Total HoNOS Score showed a positive association with each of the outcomes estimated. Without adjusting for covariates or the likelihood of HoNOS assessment, a higher HoNOS Score indicated higher overall cost (MR 1.08; 95% CI: 1.08 to 1.09), higher episode-specific costs (MR 1.09; 95% CI: 1.08 to 1.10), higher risk of relapse (OR 1.07; 95% CI: 1.07 to 1.08) and higher chance of improvement in overall scores between multiple assessments (OR 1.15; 95% CI: 1.13 to 1.16). For overall costs, weighting for the likelihood of assessment alone does not significantly change the relationship with HoNOS scores (OR 1.08; 95% CI: 1.07 to 1.09). However, adjusting for observed covariates does reduce the strength of the relationship (adj. OR 1.06; 95% CI: 1.06 to 1.06) and inverse probability weighting in addition reduces it further (adj. OR 1.05; 95% CI: 1.05 to 1.06). A similar change in the relationship between initial HoNOS Score and episode-specific cost is observed once observed covariates are adjusted for and inverse probability weights are used (adj. OR 1.04; 95% CI: 1.03 to 1.05). The same effect is not observed for relapse or improvement between assessments.

## Discussion

We conducted a comprehensive analysis of a large, real-world dataset to examine associations between patient-level and service-level factors, and the use and timing of a widely implemented routine outcome measure. Our findings demonstrate that the pattern of routine HoNOS assessment varies considerably even within a single NHS Trust, while approximately a quarter of those receiving specialist mental healthcare had no HoNOS assessment recorded. At the episode level, fewer than half of the recorded episodes of care were associated with an assessment at any point during the 6 months following the first contact with services.

Our findings strongly suggest outcome assessments are not missing at random. Multiple observed patient and service-level characteristics were strongly associated with the rates of HoNOS assessment, raising the risk of bias. Patients with more frequent or intense contact with services over longer periods of time were significantly more likely to have an assessment recorded. HoNOS assessments were more likely to be recorded for episodes with CMHTs, but repeat assessments were more likely for inpatient or specialist EIP settings. Episodes where the patient had a prior recorded diagnosis of psychosis were less likely to result in HoNOS assessment. The reason for this unexpected finding is unclear, but may reflect differing clinician priorities and practice during contact with specific patient groups. People with psychosis may be more likely to present with complex or severe difficulties compared with other mental health conditions, or require more time to achieve the aims of a care contact (eg, due to problems with communication). This may result in clinicians prioritising other tasks or interventions over completing routine outcome measures. Taken together, these findings suggest that outcome data may either systematically over-represent or under-represent those with specific (and possibly more severe) mental health conditions.

It is likely that the pattern of missingness in HoNOS scores also depends on unobserved aspects of how these assessments are used—critically, whether clinically relevant outcomes themselves (such as symptom severity, likelihood of improvement or other unobserved needs) drive patterns of assessment, for example, through financial incentives.^13^ Clinicians may be more likely to record assessments to demonstrate a need for a change in treatment, additional intervention from other professionals within the same service (eg, a psychologist or social worker) or ongoing high-intensity input—situations more commonly encountered in specific settings or services. Alternatively, assessment may be disproportionately used as an indicator that patients are ready to be discharged and used less otherwise.

The key limitation of this study was the imputed linking of outcome assessments to referrals and care contacts by date. HoNOS assessments were not always entered into the system on the date they were conducted, and in some cases appeared to be conducted by non-clinicians weeks after the most recent care contact, presumably based on patient notes for clustering purposes. Our linking methodology, therefore, likely overstates the coverage of routine outcome measurement in terms of episodes of care, as HoNOS assessments were allowed to be linked to more than one overlapping episode. This highlights the challenge of directly linking outcome assessments to the care provided, as patients with severe mental illness often receive care from multiple services simultaneously or in rapid succession, making it difficult to attribute changes in outcome measures to specific interventions, services or events. The study was also conducted on a population that is not nationally representative: only 8.4% of individuals in the study lived in the most deprived 20% of areas nationally and 89.8% of those with recorded ethnicity were identified as white individuals, compared with 81.7% nationally.^14^ Rates of HoNOS collection may be lower in more deprived catchment areas, and the patterns of missing outcome data may be different. Data quality was also a challenge in this study: ethnicity, residence and ICD-10 diagnosis were all recorded for only part of the sample, and in the case of residence and diagnosis, not always systematically or reliably updated.

The statistical drivers of HoNOS assessment and the resulting relationship between HoNOS scores and other health outcomes may not be directly applicable to other NHS Trusts or different mental healthcare systems. Other programmes of routine data collection, such as the new strategy for outcome assessment proposed by NHS England^1^ primarily using PROMs, will result in different patterns of assessment—however, as clinicians will still be involved in inviting and prompting service users to complete these, we expect some of the statistical drivers of incomplete data to be comparable. We would expect the methods used here to correct for missing outcome data to have wider relevance, but these results also highlight the importance of understanding the unique pattern of missing data in each setting. There is an increasing trend towards using real-world evidence in economic evaluations, despite the associated methodological challenges and risks of bias.^15^ Future research should focus on developing methods for reliable estimates of quality and need at the mental health service level using partially collected outcome data. In terms of HoNOS itself, the extensive data collected by the NHS and other mental health services over recent years is likely to be of continued interest. It has previously been used to assess the cost-effectiveness of EIP services,^16^ estimate the real-world treatment effects of injectable antipsychotics^17^ and monitor variation in service quality.^18 19^ Researchers and policy-makers are likely to continue using routinely collected HoNOS scores while the new system of outcome measures is being rolled out and should use appropriate care when doing so. Unlike HoNOS, the new strategy for routine patient-reported outcome measures (PROMs) will not be directly linked to payments for the provision of care. While this may eliminate incentives that potentially bias patterns of assessment and reporting, it could also remove overall incentives for routine outcome data collection and reduce the already low availability of data for service evaluation and planning. Maintaining high assessment rates without direct financial incentives while ensuring that any financial incentives that are introduced (eg, based on rates of PROM completion or improvement in PROMs) do not undermine the statistical integrity of the data collected should be an ongoing focus of implementation strategies and research.^20^

## Clinical implications

Routine outcome collection must be substantially improved for new NHS strategies to yield valid and unbiased data. Complete and consistent collection of outcome measures is crucial when using routine outcome data to monitor, evaluate, improve and commission secondary mental healthcare. However, since perfect coverage may never be feasible, robust methods for bias adjustment should be integral to service-level analyses. Further research is needed to understand why routine outcome recording may differ between specific patient groups (eg, for people with psychosis) to avoid potentially discriminatory practices. As well as improving implementation and uptake of routine outcome assessment in community mental healthcare settings, policy-makers should ensure methods are in place to produce more reliable and informative service-level aggregate measures.

## Supplementary material

10.1136/bmjment-2025-301669 online supplemental file 1

## Data Availability

No data are available.
