# Supplementary material for: Risk of bias in routine mental health outcome data: the case of Health of the Nation Outcome Scales
Source: BMJ Ment Health. 2025 Jun 5;28(1):e301669. doi: 10.1136/bmjment-2025-301669 (PMC12142120; doi:10.1136/bmjment-2025-301669)
Supplement: online supplemental file 1 [file bmjment-28-1-s001.docx]

Risk of bias in routine mental health outcome data: the case of Health of the Nation Outcome Scales – Supplementary material

# Additional tables

Table A1. Unadjusted and adjusted association of HoNOS scores with subsequent overall cost

|  | Unadjusted coefficient [95% CI] | Inverse probability weighted (IPW) coef. [95% CI] | Adjusted coef. [95% CI] | IPW and adjusted coef. [95% CI] |
| --- | --- | --- | --- | --- |
| (Intercept) | 1693.18 *** | 2341.64 *** | 991.13 *** | 1017.06 *** |
|  | [1573.22, 1822.28] | [2124.34, 2581.17] | [878.26, 1118.51] | [847.77, 1220.17] |
| Total HoNOS score | 1.08 *** | 1.08 *** | 1.06 *** | 1.05 *** |
|  | [1.08, 1.09] | [1.07, 1.09] | [1.06, 1.06] | [1.05, 1.06] |
| Location (ref: CMHT) |  |  |  |  |
| CRHT |  |  | 1.47 *** | 1.47 *** |
|  |  |  | [1.35, 1.60] | [1.35, 1.61] |
| EIP |  |  | 2.30 *** | 2.27 *** |
|  |  |  | [2.11, 2.50] | [2.08, 2.48] |
| Inpatient |  |  | 6.68 *** | 6.66 *** |
|  |  |  | [6.25, 7.14] | [6.19, 7.16] |
| PLS |  |  | 2.82 *** | 2.81 *** |
|  |  |  | [2.15, 3.70] | [2.19, 3.60] |
| Resident in most deprived quintile |  |  | 0.88 * | 0.87 * |
|  |  |  | [0.79, 0.99] | [0.77, 0.98] |
| Prior psychosis |  |  | 1.98 *** | 2.01 *** |
|  |  |  | [1.83, 2.14] | [1.82, 2.21] |
| Ethnicity (ref: BAME) |  |  |  |  |
| Not known |  |  | 0.46 *** | 0.44 *** |
|  |  |  | [0.41, 0.50] | [0.37, 0.52] |
| Other |  |  | 0.87 | 0.73 |
|  |  |  | [0.68, 1.11] | [0.50, 1.07] |
| White |  |  | 0.79 *** | 0.77 *** |
|  |  |  | [0.72, 0.87] | [0.68, 0.86] |
| Age (ref: 24 and under) |  |  |  |  |
| 25-34 |  |  | 1.13 ** | 1.23 ** |
|  |  |  | [1.04, 1.23] | [1.07, 1.42] |
| 35-44 |  |  | 1.08 | 1.14 * |
|  |  |  | [1.00, 1.18] | [1.02, 1.26] |
| 45-54 |  |  | 1.39 *** | 1.40 *** |
|  |  |  | [1.27, 1.52] | [1.26, 1.56] |
| 55-64 |  |  | 1.75 *** | 1.96 *** |
|  |  |  | [1.58, 1.94] | [1.72, 2.22] |
| 65 and over |  |  | 3.45 *** | 3.23 *** |
|  |  |  | [3.13, 3.81] | [2.79, 3.73] |
| Female |  |  | 1.08 ** | 1.13 ** |
|  |  |  | [1.02, 1.14] | [1.04, 1.22] |
| Year (ref: 2016) |  |  |  |  |
| 2017 |  |  | 1.02 | 1.01 |
|  |  |  | [0.93, 1.12] | [0.92, 1.12] |
| 2018 |  |  | 1.06 | 1.09 |
|  |  |  | [0.97, 1.17] | [0.93, 1.28] |
| 2019 |  |  | 1.14 ** | 1.12 * |
|  |  |  | [1.04, 1.25] | [1.01, 1.24] |
| 2020 |  |  | 1.31 *** | 1.34 *** |
|  |  |  | [1.20, 1.44] | [1.19, 1.50] |
| 2021 |  |  | 1.31 *** | 1.42 *** |
|  |  |  | [1.19, 1.45] | [1.27, 1.59] |
| 2022 |  |  | 1.29 | 1.25 |
|  |  |  | [0.93, 1.81] | [0.94, 1.67] |
| N | 36851 | 36851 | 36851 | 36851 |
| AIC | 673205.00 | 1384200.92 | 655111.33 | 1342176.31 |
| BIC | 673230.55 | 1384226.46 | 655315.68 | 1342380.66 |
| Pseudo R2 | 0.13 | 0.22 | 0.47 | 0.75 |

Standard errors are heteroskedasticity robust and clustered by patient. *** p < 0.001; ** p < 0.01; * p < 0.05. Outcome variable is costs for all recorded mental health contacts, inpatient admissions and bed days in the 6 months following any HoNOS assessment. Costs were estimated as a Gamma regression with a log link. Total Health of the Nation Outcome Scales (HoNOS) score is the sum of all HoNOS subscales with values for missing items imputed using non-missing values. CMHT: Community Mental Health Team. CRHT: Crisis Resolution and Home Treatment. EIP: Early Intervention in Psychosis. PLS: Psychiatric Liaison Service. BAME: Black, Asian and minority ethnic.

Table A2. Unadjusted and adjusted association of initial HoNOS scores with subsequent episode-specific cost

|  | Unadjusted coefficient [95% CI] | Inverse probability weighted (IPW) coef. [95% CI] | Adjusted coef. [95% CI] | IPW and adjusted coef. [95% CI] |
| --- | --- | --- | --- | --- |
| (Intercept) | 423.56 *** | 784.18 *** | 441.38 *** | 564.54 *** |
|  | [390.67, 459.23] | [560.90, 1096.35] | [388.83, 501.03] | [445.27, 715.76] |
| Total HoNOS score | 1.09 *** | 1.09 *** | 1.06 *** | 1.04 *** |
|  | [1.08, 1.10] | [1.06, 1.11] | [1.05, 1.06] | [1.03, 1.05] |
| Location (ref: CMHT) |  |  |  |  |
| CRHT |  |  | 0.84 *** | 0.89 ** |
|  |  |  | [0.78, 0.91] | [0.82, 0.97] |
| EIP |  |  | 1.99 *** | 1.95 *** |
|  |  |  | [1.74, 2.27] | [1.70, 2.23] |
| Inpatient |  |  | 8.34 *** | 8.88 *** |
|  |  |  | [7.15, 9.72] | [7.65, 10.31] |
| PLS |  |  | 0.27 *** | 0.27 *** |
|  |  |  | [0.23, 0.31] | [0.23, 0.31] |
| Resident in most deprived quintile |  |  | 0.96 | 1.00 |
|  |  |  | [0.87, 1.06] | [0.90, 1.12] |
| Prior psychosis |  |  | 1.40 *** | 1.42 *** |
|  |  |  | [1.25, 1.58] | [1.23, 1.64] |
| Ethnicity (ref: BAME) |  |  |  |  |
| Not known |  |  | 0.68 *** | 0.63 *** |
|  |  |  | [0.61, 0.75] | [0.51, 0.78] |
| Other |  |  | 0.99 | 0.96 |
|  |  |  | [0.74, 1.33] | [0.63, 1.47] |
| White |  |  | 0.93 | 0.79 * |
|  |  |  | [0.84, 1.03] | [0.65, 0.97] |
| Age (ref: 24 and under) |  |  |  |  |
| 25-34 |  |  | 1.10 ** | 1.12 * |
|  |  |  | [1.03, 1.16] | [1.02, 1.23] |
| 35-44 |  |  | 1.09 ** | 1.12 * |
|  |  |  | [1.02, 1.16] | [1.00, 1.24] |
| 45-54 |  |  | 1.19 *** | 1.23 *** |
|  |  |  | [1.11, 1.28] | [1.12, 1.35] |
| 55-64 |  |  | 1.26 *** | 1.36 *** |
|  |  |  | [1.16, 1.37] | [1.21, 1.53] |
| 65 and over |  |  | 3.06 *** | 2.00 *** |
|  |  |  | [2.73, 3.44] | [1.62, 2.48] |
| Female |  |  | 1.05 * | 1.08 |
|  |  |  | [1.00, 1.09] | [1.00, 1.16] |
| Year (ref: 2016) |  |  |  |  |
| 2017 |  |  | 0.98 | 0.98 |
|  |  |  | [0.92, 1.06] | [0.88, 1.08] |
| 2018 |  |  | 0.93 | 1.00 |
|  |  |  | [0.86, 1.00] | [0.88, 1.14] |
| 2019 |  |  | 0.98 | 1.07 |
|  |  |  | [0.91, 1.05] | [0.95, 1.20] |
| 2020 |  |  | 1.31 *** | 1.40 *** |
|  |  |  | [1.21, 1.42] | [1.23, 1.59] |
| 2021 |  |  | 1.36 *** | 1.34 *** |
|  |  |  | [1.25, 1.49] | [1.18, 1.52] |
| 2022 |  |  | 1.56 ** | 1.78 *** |
|  |  |  | [1.14, 2.14] | [1.35, 2.35] |
| N | 15797 | 15797 | 15797 | 15797 |
| AIC | 251897.26 | 1026535.66 | 245957.79 | 954512.28 |
| BIC | 251920.26 | 1026558.66 | 246141.81 | 954696.31 |
| Pseudo R2 | 0.18 | 0.42 | 0.44 | 0.99 |

Standard errors are heteroskedasticity robust and clustered by patient. *** p < 0.001; ** p < 0.01; * p < 0.05. Outcome variable is costs for episode-specific recorded mental health contacts, inpatient admissions and bed days in the 6 months following an initial HoNOS assessment. Costs were estimated as a Gamma regression with a log link. Total Health of the Nation Outcome Scales (HoNOS) score is the sum of all HoNOS subscales with values for missing items imputed using non-missing values. CMHT: Community Mental Health Team. CRHT: Crisis Resolution and Home Treatment. EIP: Early Intervention in Psychosis. PLS: Psychiatric Liaison Service. BAME: Black, Asian and minority ethnic.

Table A3. Unadjusted and adjusted association of HoNOS scores with subsequent relapse

|  | Unadjusted coefficient [95% CI] | Inverse probability weighted (IPW) coef. [95% CI] | Adjusted coef. [95% CI] | IPW and adjusted coef. [95% CI] |
| --- | --- | --- | --- | --- |
| (Intercept) | 0.06 *** | 0.10 *** | 0.03 *** | 0.03 *** |
|  | [0.06, 0.07] | [0.09, 0.11] | [0.03, 0.04] | [0.02, 0.04] |
| Total HoNOS score | 1.07 *** | 1.07 *** | 1.06 *** | 1.06 *** |
|  | [1.07, 1.08] | [1.06, 1.08] | [1.06, 1.07] | [1.04, 1.07] |
| Location (ref: CMHT) |  |  |  |  |
| CRHT |  |  | 34.17 *** | 27.74 *** |
|  |  |  | [30.71, 38.03] | [24.11, 31.93] |
| EIP |  |  | 1.67 *** | 1.72 *** |
|  |  |  | [1.39, 2.00] | [1.40, 2.11] |
| Inpatient |  |  | 8.70 *** | 9.00 *** |
|  |  |  | [7.53, 10.06] | [7.63, 10.62] |
| PLS |  |  | 7.26 *** | 7.88 *** |
|  |  |  | [4.80, 10.99] | [5.52, 11.25] |
| Resident in most deprived quintile |  |  | 0.79 | 0.77 |
|  |  |  | [0.63, 1.00] | [0.52, 1.14] |
| Prior psychosis |  |  | 1.13 | 1.01 |
|  |  |  | [0.99, 1.30] | [0.76, 1.34] |
| Ethnicity (ref: BAME) |  |  |  |  |
| Not known |  |  | 0.54 *** | 0.71 |
|  |  |  | [0.45, 0.64] | [0.50, 1.01] |
| Other |  |  | 0.83 | 0.61 |
|  |  |  | [0.53, 1.30] | [0.10, 3.59] |
| White |  |  | 0.79 ** | 0.91 |
|  |  |  | [0.68, 0.93] | [0.67, 1.23] |
| Age (ref: 24 and under) |  |  |  |  |
| 25-34 |  |  | 1.01 | 1.12 |
|  |  |  | [0.88, 1.16] | [0.86, 1.46] |
| 35-44 |  |  | 0.83 * | 1.02 |
|  |  |  | [0.73, 0.96] | [0.76, 1.36] |
| 45-54 |  |  | 1.12 | 1.20 |
|  |  |  | [0.97, 1.28] | [0.90, 1.60] |
| 55-64 |  |  | 1.37 *** | 1.42 * |
|  |  |  | [1.17, 1.59] | [1.04, 1.95] |
| 65 and over |  |  | 2.68 *** | 1.76 * |
|  |  |  | [2.27, 3.17] | [1.13, 2.72] |
| Female |  |  | 1.06 | 1.32 ** |
|  |  |  | [0.97, 1.15] | [1.10, 1.58] |
| Year (ref: 2016) |  |  |  |  |
| 2017 |  |  | 0.91 | 0.88 |
|  |  |  | [0.78, 1.07] | [0.65, 1.19] |
| 2018 |  |  | 1.00 | 0.89 |
|  |  |  | [0.86, 1.16] | [0.63, 1.27] |
| 2019 |  |  | 1.15 | 1.16 |
|  |  |  | [0.98, 1.33] | [0.86, 1.55] |
| 2020 |  |  | 1.09 | 1.16 |
|  |  |  | [0.93, 1.27] | [0.84, 1.61] |
| 2021 |  |  | 0.71 *** | 0.87 |
|  |  |  | [0.60, 0.83] | [0.62, 1.22] |
| 2022 |  |  | 0.34 *** | 0.33 *** |
|  |  |  | [0.19, 0.59] | [0.18, 0.60] |
| N | 36851 | 36851 | 36851 | 36851 |
| AIC | 27498.79 | 69520.50 | 20595.07 | 53339.13 |
| BIC | 27515.82 | 69537.53 | 20790.91 | 53534.97 |
| Pseudo R2 | 0.04 | 0.08 | 0.36 | 0.47 |

Standard errors are heteroskedasticity robust and clustered by patient. *** p < 0.001; ** p < 0.01; * p < 0.05. Outcome variable is relapse in the 6 months following any HoNOS assessment, where relapse is defined by referral to crisis team or inpatient admission. Relapse was estimated with logistic regression (binomial with log link). Total Health of the Nation Outcome Scales (HoNOS) score is the sum of all HoNOS subscales with values for missing items imputed using non-missing values. CMHT: Community Mental Health Team. CRHT: Crisis Resolution and Home Treatment. EIP: Early Intervention in Psychosis. PLS: Psychiatric Liaison Service. BAME: Black, Asian and minority ethnic.

Table A4. Unadjusted and adjusted association of initial HoNOS scores with subsequent HoNOS improvement

|  | Unadjusted coefficient [95% CI] | Inverse probability weighted (IPW) coef. [95% CI] | Adjusted coef. [95% CI] | IPW and adjusted coef. [95% CI] |
| --- | --- | --- | --- | --- |
| (Intercept) | 0.34 *** | 0.37 *** | 0.19 *** | 0.16 *** |
|  | [0.30, 0.40] | [0.22, 0.60] | [0.14, 0.27] | [0.07, 0.39] |
| Total HoNOS score | 1.15 *** | 1.12 *** | 1.16 *** | 1.15 *** |
|  | [1.13, 1.16] | [1.07, 1.18] | [1.14, 1.17] | [1.10, 1.21] |
| Location (ref: CMHT) |  |  |  |  |
| CRHT |  |  | 1.98 *** | 2.16 *** |
|  |  |  | [1.53, 2.57] | [1.37, 3.40] |
| EIP |  |  | 2.16 *** | 2.37 *** |
|  |  |  | [1.75, 2.65] | [1.74, 3.22] |
| Inpatient |  |  | 1.12 | 1.29 |
|  |  |  | [0.89, 1.42] | [0.83, 1.99] |
| PLS |  |  | 0.48 | 0.42 |
|  |  |  | [0.12, 1.95] | [0.08, 2.26] |
| Resident in most deprived quintile |  |  | 0.70 * | 0.67 * |
|  |  |  | [0.52, 0.94] | [0.46, 0.97] |
| Prior psychosis |  |  | 0.93 | 0.85 |
|  |  |  | [0.79, 1.09] | [0.55, 1.31] |
| Ethnicity (ref: BAME) |  |  |  |  |
| Not known |  |  | 1.53 *** | 3.81 * |
|  |  |  | [1.19, 1.97] | [1.08, 13.39] |
| Other |  |  | 1.72 | 4.12 * |
|  |  |  | [0.90, 3.27] | [1.06, 15.94] |
| White |  |  | 1.10 | 1.97 |
|  |  |  | [0.89, 1.35] | [0.65, 5.97] |
| Age (ref: 24 and under) |  |  |  |  |
| 25-34 |  |  | 0.95 | 1.11 |
|  |  |  | [0.78, 1.16] | [0.62, 1.97] |
| 35-44 |  |  | 1.13 | 0.80 |
|  |  |  | [0.91, 1.40] | [0.46, 1.39] |
| 45-54 |  |  | 0.98 | 0.64 |
|  |  |  | [0.79, 1.21] | [0.37, 1.10] |
| 55-64 |  |  | 1.06 | 0.94 |
|  |  |  | [0.84, 1.34] | [0.73, 1.22] |
| 65 and over |  |  | 1.10 | 1.12 |
|  |  |  | [0.88, 1.38] | [0.52, 2.42] |
| Female |  |  | 1.22 ** | 1.43 |
|  |  |  | [1.07, 1.40] | [0.88, 2.34] |
| Year (ref: 2016) |  |  |  |  |
| 2017 |  |  | 1.02 | 0.75 |
|  |  |  | [0.81, 1.28] | [0.44, 1.29] |
| 2018 |  |  | 1.02 | 0.81 |
|  |  |  | [0.81, 1.28] | [0.39, 1.70] |
| 2019 |  |  | 0.99 | 0.64 |
|  |  |  | [0.79, 1.24] | [0.36, 1.15] |
| 2020 |  |  | 0.86 | 0.54 |
|  |  |  | [0.68, 1.08] | [0.20, 1.46] |
| 2021 |  |  | 1.30 * | 0.68 |
|  |  |  | [1.01, 1.66] | [0.24, 1.90] |
| 2022 |  |  | 1.51 | 1.48 |
|  |  |  | [0.52, 4.42] | [0.39, 5.52] |
| N | 5195 | 5195 | 5195 | 5195 |
| AIC | 6041.51 | 119782.21 | 5925.18 | 111716.37 |
| BIC | 6054.62 | 119795.32 | 6075.95 | 111867.15 |
| Pseudo R2 | 0.17 | 0.88 | 0.21 | 0.97 |

Standard errors are heteroskedasticity robust. *** p < 0.001; ** p < 0.01; * p < 0.05. Outcome variable is any improvement in total score between two HoNOS assessments within 6 months of each other. Improvement was estimated with logistic regression (binomial with log link). Total Health of the Nation Outcome Scales (HoNOS) score is the sum of all HoNOS subscales with values for missing items imputed using non-missing values. CMHT: Community Mental Health Team. CRHT: Crisis Resolution and Home Treatment. EIP: Early Intervention in Psychosis. PLS: Psychiatric Liaison Service. BAME: Black, Asian and minority ethnic.
